# Emotion in Context: How Sender Predictability and Identity Affect Processing of Words as Imminent Personality Feedback

**DOI:** 10.3389/fpsyg.2019.00094

**Published:** 2019-02-01

**Authors:** Sebastian Schindler, Ria Vormbrock, Johanna Kissler

**Affiliations:** ^1^Department of Psychology, Bielefeld University, Bielefeld, Germany; ^2^Department of Psychology, Institute for Medical Psychology and Systems Neuroscience, University of Münster, Münster, Germany; ^3^Center of Excellence Cognitive Interaction Technology, Bielefeld University, Bielefeld, Germany

**Keywords:** EEG/ERP, social context, anticipation, emotion, language, prediction, virtual communication

## Abstract

Recent findings suggest that communicative context affects the timing and magnitude of emotion effects in word processing. In particular, social attributions seem to be one important source of plasticity for the processing of affectively charged language. Here, we investigate the timing and magnitude of ERP responses toward positive, neutral, and negative trait adjectives during the anticipation of putative socio-evaluative feedback from different senders (human and computer) varying in predictability. In the first experiment, during word presentation participants could not anticipate whether a human or a randomly acting computer sender was about to give feedback. Here, a main effect of emotion was observed only on the late positive potential (LPP), showing larger amplitudes for positive compared to neutral adjectives. In the second study the same stimuli and set-up were used, but a block-wise presentation was realized, resulting in fixed and fully predictable sender identity. Feedback was supposedly given by an expert (psychotherapist), a layperson (unknown human), and again by a randomly acting computer. Main effects of emotion started with an increased P1 for negative adjectives, followed by effects at the N1 and early posterior negativity (EPN), showing both largest amplitudes for positive words, as well as for the LPP, where positive and negative words elicited larger amplitudes than neutral words. An interaction revealed that emotional LPP modulations occurred only for a human sender. Finally, regardless of content, anticipating human feedback led to larger P1 and P3 components, being highest for the putative expert. These findings demonstrate the malleability of emotional language processing by social contexts. When clear predictions can be made, our brains rapidly differentiate between emotional and neutral information, as well as between different senders. Attributed human presence affects emotional language processing already during feedback anticipation, in line with a selective gating of attentional resources via anticipatory social significance attributions. By contrast, emotion effects occur much later, when crucial social context information is still missing. These findings demonstrate the context-dependence of emotion effects in word processing and are particularly relevant since virtual communication with unknown senders, whose identity is inferred rather than perceived, has become reality for millions of people.

## Introduction

Language processing is one of the most important and complex human abilities. We use language every day to communicate, to solace, to emote, to express happiness or sadness or to express, whether we like our counterpart or not. Specifically, emotional language is very relevant for humans since it can, sometimes just in one word, convey emotions (Barrett et al., 2007) and social evaluations. The importance of emotional language can be at least partly explained via the concept of motivated attention, implying that motivationally relevant stimuli, including emotional words, modulate attention and thus enhance perception and processing of emotional stimuli (Lang et al., 1997). The enhanced processing of emotional language can be observed using event-related potentials (ERPs), showing that words with emotional content are processed more rapidly than neutral ones and lead to amplified brain responses (e.g., see Kissler and Herbert, 2013; for reviews, see Kissler et al., 2006; Citron, 2012).

Regarding the timing of emotion effects, ERP studies show quite consistently a lager early posterior negativity (EPN) for emotional words across a variety of experimental tasks including lexical or evaluative decisions as well as passive reading (Kissler et al., 2007; Herbert et al., 2008; Scott et al., 2009; Palazova et al., 2011, 2013). The EPN arises from about 200 ms after stimulus onset and is associated with an spontaneous attention shift to emotional and arousing stimuli (Schupp et al., 2006; Kissler et al., 2009), being relatively robust against distracting tasks (Kissler et al., 2009). The fact that EPN-effects on the scalp can be observed both for intrinsically arousing and for task-relevant, explicitly attended, stimuli supports an attentional interpretation of EPN effects (Schupp et al., 2007; Schindler and Kissler, 2016b). P2 amplifications, starting around 180 ms, are also sometimes observed in emotion word processing (e.g., Kanske and Kotz, 2007; for a review, see Citron, 2012). Whether these belong to the same functional process as the EPN or are functionally entirely distinct, is currently unclear.

Emotional words have also been found to increase late parietal ERP components such as P3 and LPP. The P3 is typically associated with decision making and stimulus evaluation (Nieuwenhuis et al., 2005). The LPP is thought to be involved in memory processes and stimulus evaluation and it is further amplified by explicit and directed attention (Hajcak et al., 2009; Luck, 2012). In particular, at the late positive potential (LPP), a centro-parietal component, which arises between 400 and 800 ms after stimulus onset, emotion modulations in word processing are often observed (Hofmann et al., 2009; Schacht and Sommer, 2009; Hinojosa et al., 2010; Kissler and Herbert, 2013). Here, person-descriptive words seem to elicit stronger LPP modulations than other emotional words, suggesting that inherent socio-evaluative significance affects late processing stages (Rohr and Abdel Rahman, 2018b).

Regarding very early peaking ERP components, findings about emotion dependent modulations on the parieto-occipitally scored P1 and N1 are mixed: Some studies report early effects, while others do not observe such differentiations (for a review, see Citron, 2012). The P1 and N1 reflect early stages of stimulus detection and discrimination (Mangun and Hillyard, 1991; Hopfinger and Mangun, 1998; Vogel and Luck, 2000), and visual spatial attention can increase these ERP components (Mangun and Hillyard, 1991; Hopfinger and Mangun, 1998; Vogel and Luck, 2000; Bayer et al., 2017). A couple of studies show either valence or arousal-based modulations of such early components (Hofmann et al., 2009; Scott et al., 2009; Sass et al., 2010; Bayer et al., 2012; Keuper et al., 2013, 2014). Here, lexical frequency has been identified as an important factor: Scott et al. (2009) showed that high-frequent emotional words elicit larger N1 amplitudes than neutral words and the pattern was partly reversed for low-frequent words. Likewise, larger N1 amplitudes have been observed for high- compared to low-arousing words (Hofmann et al., 2009), while positive words seem to modulate the P1 independent of arousal (Hofmann et al., 2009; Bayer et al., 2012). For instance, when low and high arousing positive and negative words were presented in a reading task, main effects of arousal did not modulate the P1, while both categories of positive words led to a larger P1 (Bayer et al., 2012). Finally, combining EEG and MEG, very early emotion effects could be detected. These were interpreted as reflecting “emotional-tagging,” a conditioned association between emotion and words (Keuper et al., 2014). Associative learning of perceptual features has been shown to affect responses in the primary visual cortex (Rossi et al., 2017), and early ERP modulations have been recently found for pseudowords associated with reward (Bayer et al., 2018). However, other research suggests that very early emotion effects in word processing occur primarily for negative contents (Luo et al., 2010; Zhang et al., 2014). Thus, very early emotion effects in words might be restricted to particular subsets of emotional words, for recently conditioned (pseudo)words, or detectable only by very sensitive methods such as combined EEG/MEG measures, capturing both radial and tangential dipole activity.

So far, most studies investigating processing of emotional language neglected that the meaning of language is driven to a considerable extent by the communicative context in which it is embedded (Barrett et al., 2007). For instance, studies on emotional word processing show that self-relevance induced via pronouns (Herbert et al., 2011a,b) or sentence addressee can modulate early and late ERP components (Fields and Kuperberg, 2012; Bayer et al., 2017). A motivationally particularly relevant context factor in language processing is the perceived presence of a communication partner: Rohr and Abdel Rahman (2015) acoustically presented participants with the same emotional and neutral words in a communicative vs. in a non-communicative situation while recording EEG. The communicative context was created via a video of a female speaker either looking at the participant (communicative) or having her eyes closed (non-communicative). Interestingly, Rohr and Abdel Rahman observed much earlier, larger, and longer-lasting emotion effects in the communicative situation. Thus, in language processing, the timing of emotion effects seems to depend on the communicative setting.

This fits well with a recent series of studies, showing the impact of a purely psychologically constructed communicative situation on the processing of emotional words as personality feedback (Schindler et al., 2015; Schindler and Kissler, 2016a, 2018). Here, different putative senders were introduced as either another person or a computer and feedback was disclosed via color changes of positive, negative, and neutral words. Although all feedback decisions were randomly generated and counter-balanced, such that the conditions were perceptually indistinguishable, ERP enhancements for putative human feedback started with the P2 and extended throughout the EPN, P3 and LPP components. Interactions of sender and emotion regarding the feedback were observed in mid-latency time windows, showing stronger modulations of emotional feedback for human senders at the EPN or P3 component. Main effects of emotional content appeared with the EPN (Schindler and Kissler, 2016a) or P3 (Schindler et al., 2015), but the timing of the emotion effects at the feedback stage cannot be reliably interpreted, since word content had been disclosed before feedback onset. What can be said is that, following feedback disclosure, emotional content (positive or negative) is (re-)processed more strongly than neutral content and that it interacts with putative social context in that the re-processing is stronger when the “sender” is perceived as human.

While disclosure of (personality-)feedback elicits distinct and pronounced brain responses, several ERP studies point to biased stimulus processing already when anticipating socially salient events: Social relevance seems to motivate either defensive or approach-related activations: Participants who prepared to give a public speech experienced an increased level of social anxiety, and at the same time exhibited both higher physiological arousal and differential ERP responses toward angry faces, reflected in larger EPN amplitudes (Cornwell et al., 2006; Wieser et al., 2010). On the other hand, anticipating future interactions led to an enhanced processing of happy faces (Bublatzky et al., 2014; Bublatzky and Alpers, 2017). Taken together, these studies suggest that the contextual embedding modulates processing of visual stimuli already when they are about to become socially relevant. So far, only one study investigated to what extent this occurs during social feedback anticipation and if putative social context affects the timing of emotion effects (Schindler et al., 2014). This study analyzed word processing in the feedback anticipation phase of one of the aforementioned verbal virtual interaction studies, where adjectives were presented, on which a putative “human” or “computer” sender was about to give feedback. The feedback (color-change) indicated that the respective adjective was or was not descriptive for the participant. During the feedback anticipation phase, a lager N1 amplitude for a putative human sender compared to a randomly acting computer was observed. Importantly, Schindler et al. (2014) also observed interactions in the EPN and LPP time window, resulting in larger amplitudes for emotional words from the human sender. Finally, a relatively late main effect of emotion was present in the LPP as well, albeit with a more fronto-central distribution. This suggests that the anticipation of more socially relevant feedback (human sender) leads to enhanced processing of emotional words as imminent feedback. The pattern of results in this study also suggests that at least during feedback anticipation, social context appraisal precedes and tunes emotional content processing: Emotional content effects are larger in seemingly more relevant context, at least when context is already available when content is presented. This is conceptually in agreement with Rohr and Abdel Rahman’s findings.

Here, we aim to extend Schindler et al.’s (2014) finding, investigating the timing of emotion effects in predictable and unpredictable social communicative context. To shed light upon the question of how the timing of emotion effects is affected by the social communicative context and in order to detect main effects of anticipating feedback from known senders, we present two ERP studies, using the same stimuli and overall set-up, but varying in context predictability. We further explore how the words’ emotional content interacts with attributed communicative context, i.e., in the present experiment the subjective representation of a feedback sender. To this end, participants were shown positive, neutral and negative adjectives and they anticipated human or computer feedback, either under unpredictable or fixed sender-assignments. In Study 1, participants were told that they would be evaluated by a human or a randomly acting computer. However, trait adjectives were first presented, but the sender information (human/computer) was displayed simultaneously with the feedback decision (color-change), about 1.5 s into word presentation, such that the context remained initially unspecified (see Schindler and Kissler, 2018 for the effects of feedback disclosure). In Study 2, participants expected to receive feedback from an expert (therapist), a layperson, or randomly acting computer program. Here, sender-assignments were fixed across three different, counterbalanced, blocks. Thus, during adjective processing, in Study 1 no expectations about the sender could be formed, while sender-assignment and thus the social context was known beforehand in Study 2 and contextual social relevance graded, with anticipation of expert feedback arguably being more relevant than anticipation of layperson feedback and computer feedback least relevant. Please note, that the two studies were not designed *a priori* to enable statistical between-study comparisons, similar to other studies on contextual socio-emotional influences on language (Rohr and Abdel Rahman, 2018a). However, based on previous research, for each individual study clear hypotheses can be stated.

In Study 1, where the sender is unpredictable, upon word presentation, we expect typical emotion-word effects, most likely in the EPN and LPP, while no sender main effects or sender by emotion interactions should occur (unpredictable sender assignment). In Study 2, in line with Rohr and Abdel Rahman (2015), who showed remarkably early (starting already around 100 ms) and considerably larger emotion effects when emotional words were embedded in a relevant communicative context, earlier emotion effects could occur when anticipating more socially relevant feedback (human expert or layperson versus computer). Thus, during anticipation of feedback from more relevant senders we might find already P1/N1 modulations by emotional content. Based on a similar previous study (Schindler et al., 2014), we expect typical emotion effects in the EPN and LPP. Also similar to previous findings (Schindler et al., 2014), early sender effects might reflect rapid attention orienting toward stimuli associated with socially relevant feedback senders. Finally, interactions between sender and emotion should occur at the EPN and LPP level, leading to a stronger differentiation between emotional and neutral words for putative human senders. Additional effects of ascribed expertise (psychotherapist vs. layperson) are explored. Here, based on previous research, we expect larger effects for the “psychotherapist” who represents the more relevant sender context.

## Materials and Methods

### Participants

#### Study 1

For Study 1, 30 participants were recruited at Bielefeld University (20 females), who were 23 years on average (*SD* = 3.31). Participants received 11 Euro for participation.

#### Study 2

For Study 2, 39 participants were tested. Here, three participants were excluded due to large artifacts. One session had to be aborted due to a fire alarm, one participant was excluded due to a *post hoc* reported acute neurologic/psychiatric disorder, and one reported to be confused about the condition-run assignment. The resulting 33 participants (26 females) were 21.91 years of age on average (*SD* = 3.57). Participants were paid 14 Euros. Note that the second experiment took longer than the first one.

Upon structured Interview, none of the included participants reported a previous or current neurological or psychiatric disorder. All participants were right-handed and had normal or corrected-to normal vision and provided written informed consent according to the Declaration of Helsinki. The studies were approved by the Ethics Committee of Bielefeld University.

### Stimuli

In the two studies, the stimulus set of Schindler and Kissler (2018) was used. These adjectives were rated beforehand by 22 students, who did not participate in the ERP experiments, in terms of valence and arousal using the Self-Assessment Manikin (Bradley and Lang, 1994). Concreteness was also rated, using a scale that was designed in analogy to the SAM. Raters were instructed to consider the adjectives’ valence and arousal in an interpersonal evaluative context. The selected 180 adjectives (70 negative, 40 neutral, 70 positive) were matched in their linguistic properties, such as word length, frequency, familiarity, and regularity (see Table 1). Neutral adjectives were allowed to deviate from emotional adjectives on rated concreteness since truly neutral trait adjectives are rare in an interpersonal evaluative context.

**Table 1:** Comparisons of negative, neutral, and positive adjectives by one-way-ANOVAs.

Variable	Positive adjectives (*n* = 70)	Neutral adjectives (*n* = 40)	Negative adjectives (*n* = 70)	*F*_(2,147)_
Valence	7.34^a^ (0.63)	4.94^b^ (0.28)	2.85^c^ (0.67)	1016.25^***^
Arousal	4.66^a^ (0.76)	3.2^b^ (0,82)	4.78^a^ (0.74)	60.96^***^
Concreteness	2.86^a^ (1.01)	5.11^b^ (1.51)	3.18^a^ (0.66)	65.70^***^
Word length	9.30 (2.94)	8.95 (2.43)	8.79 (2.65)	0.64
Word frequency (per million)	493.69 (780.45)	512.60 (703.15)	483.43 (769.05)	0.02
Familiarity (absolute)	39934.16 (17585.69)	23488.33 (10506.85)	30036.70 (14497.37)	0.59
Regularity (absolute)	265.70 (423.44)	103.85 (186.28)	208.61 (406.98)	2.35
Neighbors Coltheart (absolute)	4.60 (6.54)	2.38 (2.95)	3.21 (3.85)	2.88
Neighbors Levenshtein (absolute)	7.47 (8.31)	4.70 (3.73)	5.86 (6.06)	2.38

*^∗∗∗^p ≤ 0.001. Standard deviations appear in parentheses below means; means in the same row sharing the same superscript letter do not differ significantly from one another at p ≤ 0.05; means that do not share subscripts differ at p ≤ 0.05 based on LSD test post hoc comparisons.*

### Procedure

The procedure was highly similar to previous studies (Schindler et al., 2015, 2018a; Schindler and Kissler, 2016a). Participants were told that they would be either evaluated by an unknown other person or randomly by a computer algorithm. Upon arrival, participants were asked to briefly describe themselves in a structured interview in front of a camera. They were informed that the video of their self-description would be presented to a human judge to give them an impression of the participant. For characterization of the sample, participants also completed a demographic questionnaire. To enhance credibility, a research assistant left the testing room 15 min ahead of the fictitious feedback, guiding either an unknown other person (Study 1) or an expert or a layperson (Study 2) to a laboratory room next to the testing room.

Stimuli were presented by software, putatively allowing instant online communication, described as “Interactional Behavioral Systems.” In order to enhance credibility of the cover story, there were network cables attaching the computer to the network, and changes of the fictitious software showing the start of possible online communication of the “Interactional Behavioral Systems” software environment were made salient. For both studies, overall, 70 positive, 40 neutral, and 70 negative words were presented. From these adjectives, 40 positive and negative adjectives were endorsed, leading to 40 affirmative negative, 40 neutral and 40 affirmative positive feedback decisions. From the adjective list, the previously rated 10 most negative and 10 most positive adjectives were always rejected to ensure face-validity (e.g., brutal, visionary). The presented feedback was randomly generated in all conditions. The desktop environment and stimulus presentation were created using Presentation^1^.

#### Study 1

In Study 1, trial-wise presentation was used. In both conditions, color changes occurred between 1,500 and 2,500 ms after word onset indicated a decision by the supposed interaction partner. We counterbalanced two colors (orange and purple) and two intensities (bright and dark) to present the feedback. In order not to confuse participants, either purple or orange were used for the ‘human’ and either the dark or the bright colors meant ‘affirmative.’ An extensive demonstration of how the software worked was shown to the participants to reduce eye-movements.

#### Study 2

In Study 2, a blocked design was used, where within each block sender assignment was clear. Sequence of putative expert and layperson sender conditions was counterbalanced across participants. In the ‘human’ conditions, color changes between 1,500 and 2,500 ms after adjective onset indicated a decision by the supposed interaction partner, while decisions from the computer varied between 1,400 and 1,600 ms. In all conditions color changes lasted for 1,000 ms, followed by a fixation cross for 1,000 to 1,500 ms. The decision was indicated via color change (blue or purple) of the presented adjective, indicating whether or not the respective adjective applied to the participant. Color–feedback assignments were counterbalanced in all conditions.

### EEG Recording and Analyses

EEG was recorded from 128 BioSemi active electrodes^2^ at 2,048 Hz. During recording, Cz was used as reference electrode. Four additional electrodes (EOG) measured horizontal and vertical eye movement, near the outer canthi of the eyes and below the eyes.

Pre-processing and statistical analyses were performed using BESA^3^ and EMEGS (Peyk et al., 2011). Offline, data was re-referenced to an average reference and a forward 0.1 Hz high-pass and a zero-phase 30 Hz low-pass filter were applied. Filtered data were segmented from 100 ms before stimulus onset until 1,000 ms after stimulus presentation. The 100 ms before stimulus onset were used for baseline correction. Eye-movements were corrected using the automatic eye-artifact correction method implemented in BESA (Ille et al., 2002). Additionally, trials exceeding a threshold of 120 μV were rejected. For Study 1, overall, 6.25% of all electrodes were interpolated. On average, 13.85% of all trials were rejected. There were no differences in the number of rejected trials in regard of sender [*F*_(1,29)_ = 0.19, *p* = 0.663, ${\text{η}}_{\text{p}}^{2}$ = 0.007], emotional content [*F*_(2,58)_ = 1.11, *p* = 0.337, ${\text{η}}_{\text{p}}^{2}$ = 0.037], or an interaction [*F*_(2,58)_ = 0.52, *p* = 0.600, ${\text{η}}_{\text{p}}^{2}$ = 0.017]. In Study 2, overall, 5.16% of all electrodes were interpolated. On average, 13.50% of all trials were rejected. There were no differences in the number of rejected trials in regard of sender [*F*_(2,64)_ = 0.61, *p* = 0.511, ${\text{η}}_{\text{p}}^{2}$ = 0.019], emotional content [*F*_(2,64)_ = 1.06, *p* = 0.351, ${\text{η}}_{\text{p}}^{2}$ = 0.032], or an interaction [*F*_(4,128)_ = 1.06, *p* = 0.371, ${\text{η}}_{\text{p}}^{2}$ = 0.032].

### Statistical Analyses

**FIGURE 1:**
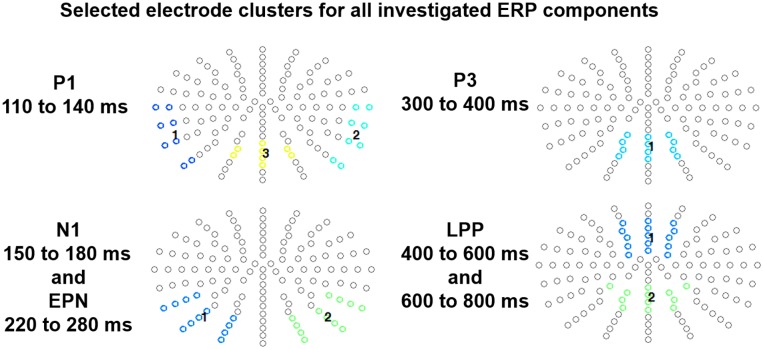
Selected electrode clusters for each ERP component. Each color indicates a respective electrode cluster.

EEG scalp data were statistically analyzed with EMEGS. For Study 1, 2 (sender: human, computer) × 3 (content: positive, neutral, negative), for Study 2, 3 (sender: human expert, computer, human layperson) × 3 (content: positive, neutral, negative), repeated-measures ANOVAs investigated time windows and electrode clusters of interest. Partial eta-squared (partial η^2^) was estimated to describe effect sizes (Cohen, 1988). Due to the imbalance of the sender numbers in the two studies, no direct statistical between-study comparisons were calculated. When Mauchly’s test indicated a violation of sphericity, degrees of freedom were corrected according to Greenhouse–Geisser. For readability, uncorrected degrees of freedom are reported, while *p*-values and effect sizes are corrected. For significant main effects and interactions, *post hoc* comparisons using Fisher’s Least Significant Difference test were set up to investigate the direction of the observed differences. Time windows and electrode clusters for components of interest were based on previous studies. For P1, N1, EPN, and LPP analyses, region was included as a factor (P1 left/mid/right; N1 and EPN left/right; LPP anterior/posterior)^4^ and analyses focused on a single window for each component, while for the LPP time (early/late) was included into the OMNIBUS analyses. For the P1, time windows were segmented from 110 to 140 ms, scoring the P1 with two lateral temporo-occipital clusters (e.g., see Hofmann et al., 2009; Kissler et al., 2009) and a central parieto-occipital cluster (Herbert et al., 2008; Scott et al., 2009; Sass et al., 2010; Schindler et al., 2014) of same sizes (left cluster eight electrodes: T9, T7, TP9, TP7, P9, P7, PO9, PO7; central cluster eight electrodes: PPOz, PO3, POz, PO4, POO3, POOz, POO4, Oz; right cluster eight electrodes: T10, T8, TP10, TP8 P10, P8, PO10, PO8; see Figure 1). From 150 to 180 ms the N1, and from 220 to 280 ms the EPN was scored (similar in time and regions to various word studies, e.g., see Kissler et al., 2007; Hofmann et al., 2009; Scott et al., 2009; Kissler and Herbert, 2013; Schindler and Kissler, 2016a, 2018), applying two symmetrical occipital clusters of 12 electrodes each (left: I1, OI1, O1, PO9, PO9h, PO7, P9, P9h, P7, POO3, PO5, P5; right: I2, OI2, PO10, PO10h, PO8, P10, P10h, P8, POO4, PO6, P6). Further, the P3 was identified from 300 to 400 ms, using a parietal cluster (12 electrodes: P1, Pz, P2, PP01, PPOz, PPPO2, PO3, POz, PO4, POO3, POOz, POO4). Finally, for the LPP, based on previous similar studies (e.g., see Kissler et al., 2009; Solomon et al., 2012; Schindler et al., 2014; Klein et al., 2015), an early (400–600 ms) and late (600–800 ms) portion was distinguished, using an anterior and posterior cluster (anterior cluster of 15 electrodes: AF1, AFz, AF2, AFF1, AFFz, AFF2, F1, Fz, F2, FFC1, FFCz, FFC2, FC1, FCz, FC2; Posterior cluster of 12 electrodes: CPP3h, CPPz, CPP4h, P1, Pz, P2, PP01, PPOz, PPPO2, PO3, POz, PO4).

## Results

### Study 1

#### P1

**FIGURE 2:**
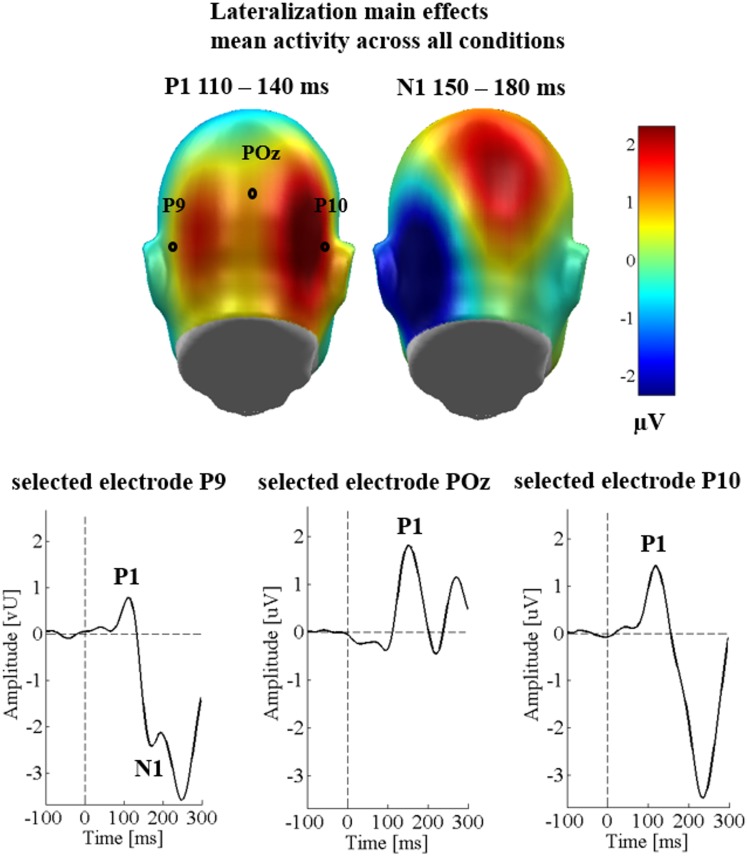
Lateralization effects in Study 1. Upper panel shows scalp topographies for all conditions collapsed. Blue color indicates more negativity and red color more positivity. Lower panel shows selected electrodes for each of the P1 clusters.

Over the three occipital sensor clusters, no main effects of emotion [*F*_(2,58)_ = 1.42, *p* = 0.251, ${\text{η}}_{\text{p}}^{2}$ = 0.047], the communicative sender [*F*_(1,29)_ = 0.59, *p* = 0.451, ${\text{η}}_{\text{p}}^{2}$ = 0.020], and no significant interaction between sender and emotion were found [*F*_(2,58)_ = 0.014, *p* = 0.986, ${\text{η}}_{\text{p}}^{2}$ < 0.001]. There was a main effect of region [*F*_(2,58)_ = 6.04, *p* = 0.004, ${\text{η}}_{\text{p}}^{2}$ = 0.172; see Figure 2], showing larger P1 amplitudes over the right temporo-occipital cluster compared to the left temporo-occipital cluster (*p* = 0.008), and compared to the parieto-occipital cluster (*p* = 0.004). The latter two clusters did not differ from each other (*p* = 0.340). All other possible interactions were insignificant (*F*s < 1.76; *p*s > 0.143).

#### N1

Over the occipital sensor clusters, again no main effects of emotional content [*F*_(2,58)_ = 1.47, *p* = 0.239, ${\text{η}}_{\text{p}}^{2}$ = 0.048], sender [*F*_(1,29)_ = 0.17, *p* = 0.682, ${\text{η}}_{\text{p}}^{2}$ = 0.006], and no interaction of sender with emotion [*F*_(2,58)_ = 0.23, *p* = 0.798, ${\text{η}}_{\text{p}}^{2}$ = 0.008] were observed. There was a large main effect of region [*F*_(1,29)_ = 23.97, *p* < 0.001, ${\text{η}}_{\text{p}}^{2}$ = 0.453], showing stronger N1 amplitudes over the left sensor cluster (see Figure 2). All other possible interactions were insignificant (*F*s < 2.81; *p*s > 0.104).

#### EPN

For the EPN, between 220 and 280 ms, no main effects of emotional content [*F*_(2,58)_ = 0.21, *p* = 0.809, ${\text{η}}_{\text{p}}^{2}$ = 0.007], sender [*F*_(1,29)_ = 1.46, *p* = 0.235, ${\text{η}}_{\text{p}}^{2}$ = 0.048], and no interaction of sender with emotion [*F*_(2,58)_ = 2.63, *p* = 0.080, ${\text{η}}_{\text{p}}^{2}$ = 0.083] were observed. Neither was there a main effect of region [*F*_(1,29)_ = 2.16, *p* = 0.125, ${\text{η}}_{\text{p}}^{2}$ = 0.069], nor were any interactions significant (*F*s < 2.43; *p*s > 0.130).

#### P3

For the P3 component, between 300 and 400 ms, no main effects of emotion [*F*_(2,58)_ = 0.50, *p* = 0.610, ${\text{η}}_{\text{p}}^{2}$ = 0.017], of the communicative sender [*F*_(1,29)_ = 0.37, *p* = 0.546, ${\text{η}}_{\text{p}}^{2}$ = 0.013], and no significant interaction between sender and emotion were found [*F*_(2,58)_ = 0.71, *p* = 0.466, ${\text{η}}_{\text{p}}^{2}$ = 0.024].

#### LPP

**FIGURE 3:**
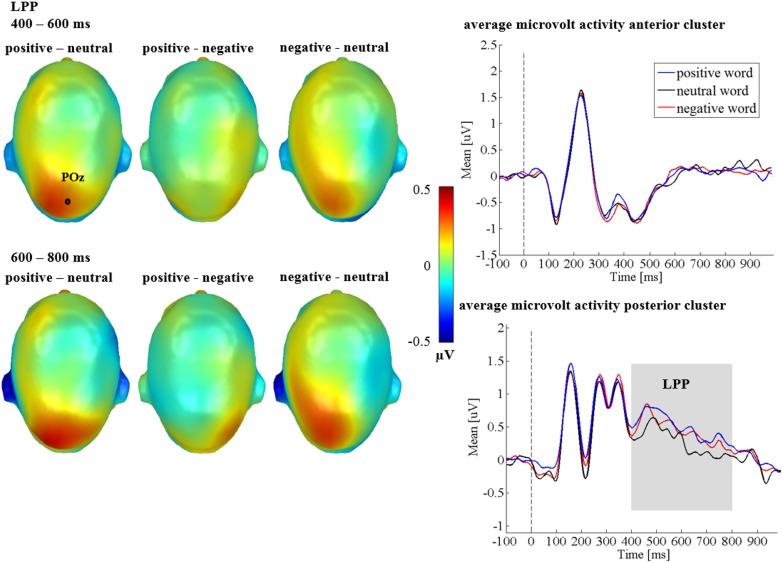
Emotion LPP effects in Study 1. Left panel depicts difference topographies between emotional contents. Blue color indicates more negativity and red color more positivity for the respective comparison. Right panel: Average amplitudes for the anterior and posterior electrode clusters, showing the time course for all conditions. Time windows with significant effects are highlighted in gray.

For the LPP, a main effect of emotion was found [*F*_(1.54,44.72)_ = 3.69, *p* = 0.044, ${\text{η}}_{\text{p}}^{2}$ = 0.113; see Figure 3], showing more positive LPP amplitudes for positive adjectives compared to neutral adjectives (*p* = 0.033), while neither negative and neutral adjectives (*p* = 0.073), nor positive and negative adjectives (*p* = 0.520) differed significantly. Although the emotion by region interaction was insignificant [*F*_(1.55,44.81)_ = 1.54, *p* = 0.223, ${\text{η}}_{\text{p}}^{2}$ = 0.050], descriptively the emotion effect seemed to be stronger over the posterior cluster (see Figure 3). There was no main effect of the communicative sender [*F*_(1,29)_ = 0.441, *p* = 0.512, ${\text{η}}_{\text{p}}^{2}$ = 0.015], and no significant interaction between sender and emotion was found [*F*_(2,58)_ = 0.73, *p* = 0.487, ${\text{η}}_{\text{p}}^{2}$ = 0.024]. A main effect of region [*F*_(1,29)_ = 9.63, *p* = 0.004, ${\text{η}}_{\text{p}}^{2}$ = 0.249] showed that amplitudes over the posterior cluster were larger than over the anterior cluster (right panel of Figure 3). Further, an interaction of time and regions [*F*_(1,29)_ = 24.23, *p* < 0.001, ${\text{η}}_{\text{p}}^{2}$ = 0.455], showed that, overall, the LPP amplitude decreased during the late LPP time window (see Figure 3, bottom right). All other possible main and interaction effects were insignificant (*F*s < 3.00; *p*s > 0.071).

### Study 2

#### P1

**FIGURE 4:**
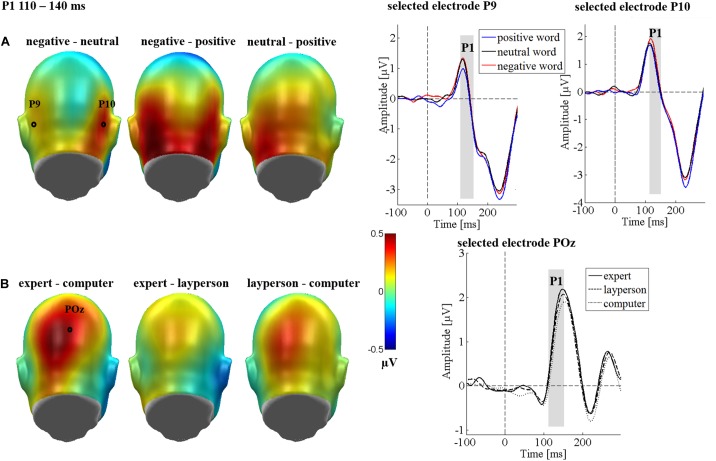
P1 main effects of the sender and emotion in Study 2. The left panel shows difference topographies: Blue color indicates more negativity and red color more positivity for the respective comparison. The right panel shows the time course for selected electrodes. **(A)** A significantly larger P1 was found for negative compared to positive words. **(B)** A significantly larger P1 was found for the expert compared to the computer sender for the central parieto-occipital electrode cluster.

Over the occipital clusters, a main effect of emotion [*F*_(2,64)_ = 3.46, *p* = 0.037, ${\text{η}}_{\text{p}}^{2}$ = 0.098; see Figure 4A] was found. Here, negative adjectives elicited a larger P1 compared to positive adjectives (*p* = 0.013; see Figure 4A), while not significantly differing from neutral adjectives (*p* = 0.054). Positive and neutral adjectives did not differ from each other (*p* = 0.395). There was no significant main effect of the communicative sender [*F*_(2,64)_ = 0.82, *p* = 0.444, ${\text{η}}_{\text{p}}^{2}$ = 0.025] and no significant interaction between sender and emotion [*F*_(4,128)_ = 0.60, *p* = 0.661, ${\text{η}}_{\text{p}}^{2}$ = 0.019]. A main effect of region [*F*_(2,64)_ = 6.15, *p* = 0.004, ${\text{η}}_{\text{p}}^{2}$ = 0.161] showed larger P1 amplitudes over the right temporo-occipital cluster compared to the left temporo-occipital cluster (*p* = 0.003), while the right temporo-occipital cluster did not differ from the parieto-occipital cluster (*p* = 0.068). Finally, the left temporo-occipital cluster and the parieto-occipital did not differ from each other either (*p* = 0.073).

An interaction of communicative sender and region was observed [*F*_(4,128)_ = 2.65, *p* = 0.049, ${\text{η}}_{\text{p}}^{2}$ = 0.077; see Figure 4B]. Here, *post hoc* tests showed that an effect of the communicative sender was observed parieto-occipitally [*F*_(2,64)_ = 3.51, *p* = 0.036, ${\text{η}}_{\text{p}}^{2}$ = 0.099; see Figure 4A], whereas no sender effects occurred over the left [*F*_(2,64)_ = 0.18, *p* = 0.982, ${\text{η}}_{\text{p}}^{2}$ = 0.001], or right electrode cluster [*F*_(2,64)_ = 0.89, *p* = 0.415, ${\text{η}}_{\text{p}}^{2}$ = 0.027]. Over the central cluster, a stronger P1 modulation was observed when anticipating expert feedback than when anticipating the computer feedback (*p* = 0.010; see Figure 4A). No significant differences were found between the expert and layperson (*p* = 0.350), or the layperson and the computer (*p* = 0.127). All other possible other interactions were insignificant (*F*s < 1.25; *p*s > 0.272).

#### N1

**FIGURE 5:**
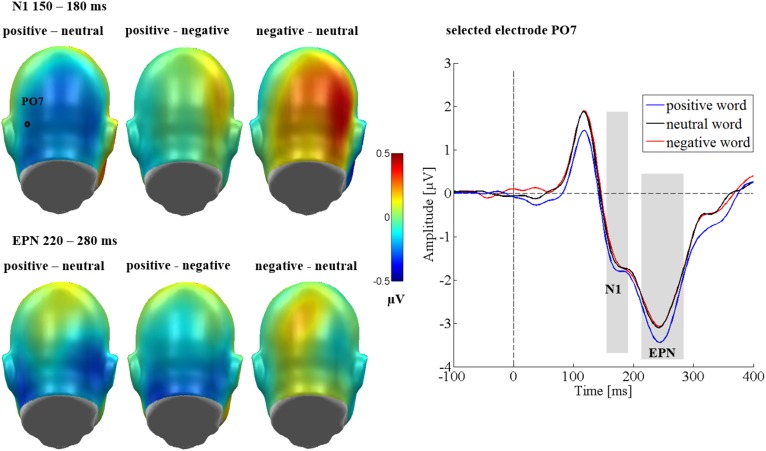
N1 and EPN occipital emotion effects in Study 2. Left panel shows difference topographies: Blue color indicates more negativity and red color more positivity for the respective comparison. A significantly larger N1 and EPN was found for positive words compared to negative ones. Right panel: Selected electrode OI2 shows the time course over occipital regions. Time windows with significant effects are highlighted in gray.

Over the occipital sensor clusters, a main effect of emotion [*F*_(2,64)_ = 3.30, *p* = 0.043, ${\text{η}}_{\text{p}}^{2}$ = 0.094; see Figure 5] was found. Here, positive words elicited a larger N1 amplitude than negative words (*p* = 0.008). Neutral words did not differ significantly from negative words (*p* = 0.065) or positive words (*p* = 0.866). Neither was there a main effect of the communicative sender [*F*_(2,64)_ = 0.37, *p* = 0.691, ${\text{η}}_{\text{p}}^{2}$ = 0.011], nor a significant interaction between sender and emotion [*F*_(4,128)_ = 0.32, *p* = 0.866, ${\text{η}}_{\text{p}}^{2}$ = 0.010]. For the N1, a large effect of region was observed [*F*_(1,32)_ = 15.62, *p* < 0.001, ${\text{η}}_{\text{p}}^{2}$ = 0.328], showing larger N1 amplitudes over the left sensor cluster. All other possible interactions were insignificant (*F*s < 1.48, *p*s > 0.235).

#### EPN

Over the occipital sensor clusters, there was a modulation of the EPN by emotional content [*F*_(2,64)_ = 4.29, *p* = 0.018, ${\text{η}}_{\text{p}}^{2}$ = 0.118, see Figure 5]. Here, positive words elicited a larger EPN amplitude compared to negative (*p* = 0.023) and neutral words (*p* = 0.008), while negative and neutral words did not differ from each other (*p* = 0.906). No effect of the communicative sender [*F*_(2,64)_ = 2.16, *p* = 0.123, ${\text{η}}_{\text{p}}^{2}$ = 0.063], and no interaction between sender and emotion [*F*_(4,128)_ = 1.21, *p* = 0.312, ${\text{η}}_{\text{p}}^{2}$ = 0.036] were found. An effect of region [*F*_(1,32)_ = 10.93, *p* = 0.002, ${\text{η}}_{\text{p}}^{2}$ = 0.255] showed larger amplitudes over the left sensor cluster. All other possible interactions were insignificant (*F*s < 0.96, *p*s > 0.434).

#### P3

**FIGURE 6:**
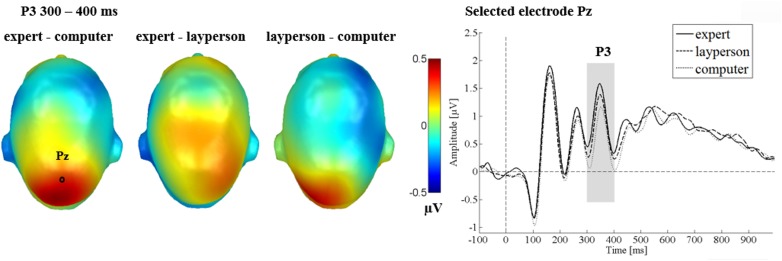
P3 main effects of the sender in Study 2. The left panel shows difference topographies: Blue color indicates more negativity and red color more positivity for the respective comparison. The right panel shows the time course for selected electrode Pz. A significantly larger P3 was found for the expert and layperson compared to the computer sender.

Centrally, for the P3 component, between 300 and 400 ms, no main effect of emotion [*F*_(2,64)_ = 1.77, *p* = 0.176, ${\text{η}}_{\text{p}}^{2}$ = 0.052] was found, while a significant main effect of the communicative sender was observed [*F*_(2,64)_ = 6.29, *p* = 0.003, ${\text{η}}_{\text{p}}^{2}$ = 0.164; see Figure 6]. When anticipating feedback from the expert (*p* = 0.002), or from the layperson (*p* = 0.050), P3 amplitudes were larger than for anticipating “computer” feedback. There were no significant differences between the expert and layperson (*p* = 0.150). Finally, no significant interactions between sender and emotion were found [*F*_(4,128)_ = 0.27, *p* = 0.896, ${\text{η}}_{\text{p}}^{2}$ = 0.008].

#### LPP

For the LPP, neither a main effect of emotion [*F*_(1.62,51.77)_ = 2.84, *p* = 0.066, ${\text{η}}_{\text{p}}^{2}$ = 0.081], nor a main effect of the communicative sender [*F*_(2,64)_ = 0.39, *p* = 0.679, ${\text{η}}_{\text{p}}^{2}$ = 0.012] were found.

**FIGURE 7:**
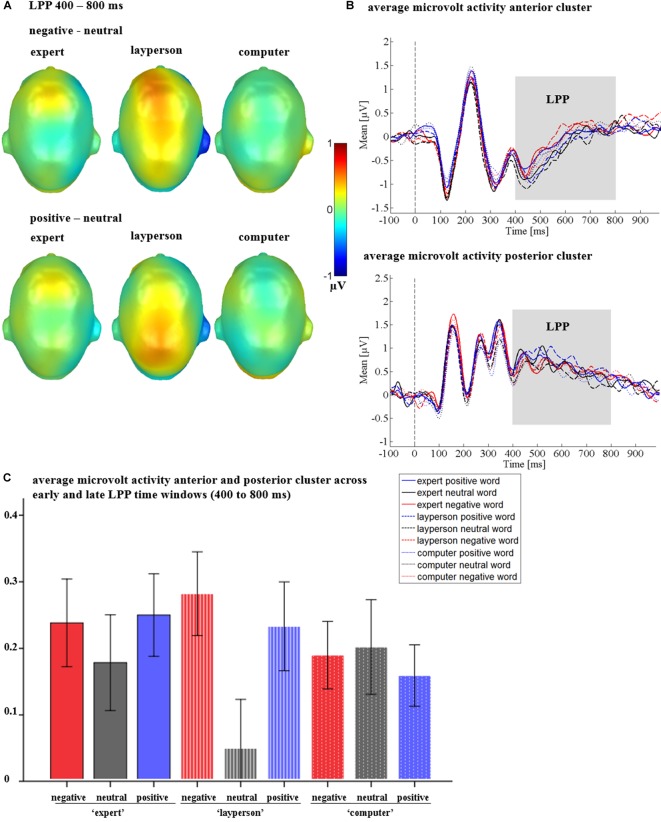
LPP interactions between sender and emotion in Study 2. **(A)** Difference topographies, showing strongest emotion to neutral differences for the layperson. **(B)** Average amplitudes in microvolt for the anterior and posterior cluster for all conditions. **(C)** Average amplitudes in microvolt for the whole LPP. Error bars represent ±1 SEM.

Crucially, a significant interaction between sender and emotion was observed [*F*_(4,128)_ = 3.92, *p* = 0.005, ${\text{η}}_{\text{p}}^{2}$ = 0.109; see Figure 7], The sender by emotion interaction showed that emotion differences were present for the layperson sender [*F*_(2,64)_ = 7.01, *p* = 0.002, ${\text{η}}_{\text{p}}^{2}$ = 0.180; see Figures 7A,C], but not within the expert [*F*_(2,64)_ = 1.26, *p* = 0.291, ${\text{η}}_{\text{p}}^{2}$ = 0.038], or the computer [*F*_(1.65,52.79)_ = 0.29, *p* = 0.710, ${\text{η}}_{\text{p}}^{2}$ = 0.009]. Within the layperson, both positive (*p* = 0.004), and negative adjectives (*p* = 0.005) elicited more positively going amplitudes than did neutral adjectives. Positive and negative adjectives did not differ from each other (*p* = 0.407).

Further, there was a significant emotion by time interaction [*F*_(2,64)_ = 4.28, *p* = 0.018, ${\text{η}}_{\text{p}}^{2}$ = 0.118], as well as a triple interaction of emotion by time by region [*F*_(2,64)_ = 4.12, *p* = 0.021, ${\text{η}}_{\text{p}}^{2}$ = 0.114]. For the emotion by time interaction, a significant emotion effect emerged in the early LPP (400 to 600 ms) [*F*_(1.68,53.80)_ = 4.04 *p* = 0.029, ${\text{η}}_{\text{p}}^{2}$ = 0.112]. Here, positive adjectives elicited a larger LPP than neutral adjectives (*p* = 0.013). Negative and neutral adjectives (*p* = 0.065) or positive and negative adjectives did not differ from each other (*p* = 0.624). At late stages (600–800 ms), no main effect of emotion was observed [*F*_(2,64)_ = 2.12, *p* = 0.128, ${\text{η}}_{\text{p}}^{2}$ = 0.062]. Regarding the triple interaction of emotion by time by region, in the early LPP, no main effect of emotion could be found posteriorly [*F*_(1.50,47.92)_ = 1.50, *p* = 0.232, ${\text{η}}_{\text{p}}^{2}$ = 0.045], instead, it was located anteriorly [*F*_(2,64)_ = 4.07, *p* = 0.022, ${\text{η}}_{\text{p}}^{2}$ = 0.113]. Here, negative adjectives differed from neutral adjectives (*p* = 0.014), while other comparisons were insignificant (*ps* > 0.110). Similar to the emotion by time interaction, at late stages (600–800 ms), emotion effects were found neither over the anterior [*F*_(2,64)_ = 0.97, *p* = 0.386, ${\text{η}}_{\text{p}}^{2}$ = 0.029], nor over the posterior cluster [*F*_(2,64)_ = 0.74, *p* = 0.482, ${\text{η}}_{\text{p}}^{2}$ = 0.023].

There was a main effect of region [*F*_(1,32)_ = 19.72, *p* < 0.001, ${\text{η}}_{\text{p}}^{2}$ = 0.381], showing that LPP amplitudes over the posterior cluster were larger than over the anterior cluster (see Figure 7B). Further, a main effect of time [*F*_(1,32)_ = 12.19, *p* = 0.001, ${\text{η}}_{\text{p}}^{2}$ = 0.276], revealed that overall, LPP amplitudes increased during the late LPP time window (600–800 ms). However, the main effects of time and region were qualified by an interaction of time and region [*F*_(1,32)_ = 33.15, *p* < 0.001, ${\text{η}}_{\text{p}}^{2}$ = 0.509], showing that the differences between posterior and anterior amplitudes decreased over time (see Figure 7B). All other possible interaction effects were insignificant (*F*s < 0.88; *p*s > 0.479).

## Discussion

In the present study, we investigate how effects of emotional content in word processing are influenced by predictable or unpredictable social contexts. We specifically explored how social communicative context affects the timing of emotion effects. Further, we tested how anticipating predictable social (“human”) compared to non-social (“computer”) feedback alters processing of the very same word, as well as possible interactions of emotional content and sender status. In two ERP studies, the same positive, neutral and negative trait adjectives were presented while participants anticipated feedback on a given adjective. As a crucial difference between the studies, participants in Study 1 were unaware of the specific sender identity and were awaiting its disclosure simultaneously with the realization of the feedback itself, while participants in Study 2 had a clear sender mapping. We hypothesized that the predictable sender information in Study 2 would affect the timing of emotion effects, as well as elicit larger attention-related early ERP amplitudes for socially relevant senders (human expert and layperson), while interactions between sender and emotion were expected at the EPN or LPP stages. Finally, in both studies emotional content was expected to affect EPN and LPP amplitudes. The occurrence of other, particularly pre-EPN, emotion effects was explored.

The discussion will focus on the (dis-)similarities of effects induced by the same materials when contextual information was available to a different extent. However, because there were two anticipated senders in Study 1 and three in Study 2, no formal statistical comparison is performed. This is similar to Rohr and Abdel Rahman’s (2015) approach who did not perform a direct between-condition comparison either, as overall ERP morphology differed considerably between their communicative and non-communicative conditions.

### Early Emotion Effects Only Occur in Predictable Social Context

In Study 1, in accordance with other studies (e.g., Kissler et al., 2009; Schacht and Sommer, 2009; Hinojosa et al., 2010; Bayer and Schacht, 2014; Schindler et al., 2018b), no very early (pre-EPN) emotional modulations were observed. However, in Study 2, where sender assignment was predictable, differential processing of emotional adjectives started with very early components modulating P1 and N1 responses. This is highly interesting, since findings on very early emotion effects are mixed (Citron, 2012). For instance, a previous study by Schindler et al. (2014) that used a very similar social context manipulation as the present Study 2, did not report such very early emotion effects. Because the 2014 study had fewer stimuli and participants, lower experimental power could have been one reason for this difference. Some other emotional language studies report very early effects only for high-arousing stimuli (Gianotti et al., 2008; Hofmann et al., 2009; Keuper et al., 2014), or for positive content (Hofmann et al., 2009; Bayer et al., 2012). Bayer et al. (2012) found increased P1 amplitudes for positive content regardless of arousal, which was interpreted to reflect approach-related motivation. Similarly, such early valence differentiations (Gianotti et al., 2008; Keuper et al., 2013), were previously interpreted as an intrinsic “pleasantness check” (e.g., the component process model of emotion, see Scherer, 2001). Other findings report a decrease of P1 amplitudes for positive words Hofmann et al. (2009) or an increase of the P1 amplitudes for negative words (Zhang et al., 2014). Based on such findings, three stages of emotional processing have been proposed, with initially amplified negative content processing, followed by an arousal processing and finally a late, more detailed, valence differentiation (Luo et al., 2010; Zhang et al., 2014). The present results from our Study 2 corroborate these findings in that we also observe very early valence differentiation, with a smaller P1 for positive compared to negative words, neutral falling between the other two. Overall, however, the literature on the direction of very early emotion effects is quite inconsistent. Possibly, the relative sensitivity of participants’ motivational approach or avoidance systems drives these very early valence differentiations. This possibility awaits formal testing, probably requiring very large samples.

From a different angle, it has been argued that such early effects are due to a conditioned association between perceptual features of the word stimulus and emotional content (Ortigue et al., 2004). Ortigue et al. (2004) found enhanced brain responses to emotional words between 100 and 140 ms, interpreted to reflect a distinct network for early emotional word processing in the right hemisphere, based on mnemonic templates. Supporting this view, early ERP modulations have been observed for pseudowords, previously associated with reward (Bayer et al., 2018). Consistent with the idea that well-learned word forms are rapidly matched with mnemonic templates, very early emotion effects on the N1 have been observed for high- but not for low-frequent material (Scott et al., 2009). This interaction has been interpreted as a marker of very early lexical access (Citron, 2012).

Indeed, emotional content has been found to accelerate lexical access in the EPN time-window (Kissler and Herbert, 2013). Crucially, however, present and previous data suggest that the timing of such emotion effects is itself influenced by the social communicative context. Rohr and Abdel Rahman (2015) found very early emotion effects (starting 100 ms after stimulus onset) in a communicative and therefore socially relevant situation. Interestingly, in the non-communicative condition, the same stimulus material evoked emotion effects not earlier than 250 ms after stimulus onset, which is conceptually similar to the present pattern across our two experiments. Predictable communicative context in our study likewise seems to accelerate the distinction between emotional content, leading to a larger P1 for negative words, while positive words elicited increased N1 and EPN amplitudes. For the LPP, which has been related to stimulus evaluation and memory formation (Herbert et al., 2008; Hofmann et al., 2009), both positive and negative words elicited a larger positivity than neutral words. In fact, in the present Study 1, emotion effects emerged in the LPP window for the first time. This is in agreement with Rohr and Abdel-Rahman’s findings on earlier, more pronounced and longer lasting emotion effects in a communicative compared to a non-communicative context (2015).

In this regard, in contrast to Study 1, Study 2 showed also an EPN modulation for emotional, and specifically positive adjectives. In the literature, enlarged EPN amplitudes for emotional words are relatively consistently shown by ERP studies (Kissler et al., 2007; Herbert et al., 2008; Scott et al., 2009; Palazova et al., 2011, 2013). The different pattern between Study 1 and Study 2 might be explained by a different focus of attention in the two studies that could affect the timing of emotion effects. In Study 1, participants might delay content processing until shortly before context disclosure, context being a crucial factor in determining affective significance. In Study 2, by contrast, sender-assignments were fixed, and emotional information could be prioritized earlier. In this vein, when emotional words were presented within sentences, no emotional EPN modulation was found, while LPP effects could be detected (Bayer et al., 2010). Here, the authors argued that the encoding of the sentences captures attention resources, so that there are less resources for stimulus-driven attention to emotional content (Bayer et al., 2010). Moreover, in sentence contexts integration of contextual information seems crucial in determining the affective significance of a sentence. Accordingly, Fischler and Bradley (2006) in their affective sentence processing studies also observe reliable fronto-central LPP modulation as the first and only emotion-driven modulation. The selective amplification of positive adjectives in Study 2 for the N1 and EPN could reflect an optimistic attentional bias, suggesting that humans are more likely to expect positive feedback in social interactions (Watson et al., 2007; Hepper et al., 2011; Sharot et al., 2011; Korn et al., 2012; Van der Molen et al., 2014).

### Late Interactions of Sender and Emotion Under Predictable Social Context

Interestingly, in Study 1, an emotion effect in the LPP time window was found, most pronounced for the posterior sensor cluster, replicating results from various previous studies, which show that the LPP is increased by emotional words (e.g., see Hofmann et al., 2009; Schacht and Sommer, 2009; Hinojosa et al., 2010; Kissler and Herbert, 2013). In Study 2, emotional LPP modulations were observed to interact with time and region. However, most importantly, they were modulated by the predictable sender. This portends to different generators of the emotional LPP in the two studies, as well as between the expert and layperson in Study 2, again supporting the notion that processing of language is driven to a considerable extent by the embedding context (Barrett et al., 2007). Interestingly, previous studies report different topographies in the LPP (e.g., see Kissler et al., 2009). Kissler et al. (2009) reported separate main effects of task and emotion effects in the LPP, the task effect showing a frontal and the emotion effect a parietal distribution. It was hypothesized, that simultaneous processes of emotion and of task, the latter presumably recruiting frontal engagement, led to different topographical patterns. Other studies showing interactions of task and emotion (Schupp et al., 2007; Schindler and Kissler, 2016b) likewise report fronto-central LPP effects. Thus, a parallel and interactive processing in Study 2 could explain such shifts in the topography between the two studies. In Study 1, unsurprisingly, no interactions between emotion and sender were observed, but in Study 2, interactions similar to the pattern observed in Schindler et al. (2014) were found. While for the expert, emotional words seem to induce a larger frontal positivity (see Figure 7A), emotional differences were only significant within the layperson, where both negative and positive words elicited a larger LPP (see Figure 7C). Finally, within the computer no valence differences were observed. A tentative explanation for this specific pattern is that anything coming from an expert is potentially relevant, regardless of content, whereas all computer feedback is generally less relevant, such that within the “layperson,” there is most room for content-driven differentiation, thereby replicating Schindler et al. (2014).

These findings in general underscore the context-dependence of emotion effects and are in line with previous studies, showing that the anticipation of socially salient events, e.g., a public speech (Cornwell et al., 2006; Wieser et al., 2010), or a future interaction, amplify the processing of negative or positive emotional content (Bublatzky et al., 2014). The present results further replicate that already anticipating feedback from a relevant human sender modulates the processing of the presented emotional words (Schindler et al., 2014). This is in line with the notion that the LPP amplitude reflects an enhanced processing of biologically relevant stimuli (Lang et al., 1997; Schupp et al., 2006), and has been extended to socio-emotional context recently (Schindler and Kissler, 2016a, 2018). Layperson (peer) feedback may activate more distributed sources, while for the expert more frontally driven networks are activated in preparation of any kind of feedback.

### Early and Late Main Effects of Predictable Social Feedback Sender

The second main question focused on the impact of predictable communicative senders on word processing. While, as expected, there were no sender effects in Study 1 as the sender was unknown, in Study 2 a main effect of the communicative sender was found for the P1 and P3 components. We explored possible differences between a supposed expert and layperson, both being introduced as human senders. Here, a larger P1 for the anticipation of feedback from the expert compared to the computer sender was found, the layperson falling between these two senders. Such an early effect of the communicative sender is in line with a previous study about the anticipation of feedback from a human sender compared to a computer sender, where sender effects were found between 100 and 150 ms after word onset (Schindler et al., 2014). The present finding could suggest that the anticipation of socially relevant feedback enhanced and directed attention to the word stimuli in a bottom up fashion. From a different angle, Klimesch (2011), suggests that the P1 could reflect an inhibitory neuronal process which is related to early access to knowledge systems, being an inhibitory feedback wave from ‘higher’ cortical areas. Thus, when regarding the P1 as the first index of attentional control interacting with bottom-up sensory processing (Klimesch et al., 2007), this indicates that a gating occurs for words becoming socially relevant. Such early anticipatory modulations were observed by Michalowski et al. (2009, 2015), who showed a higher P1 for spider phobic subjects not only in response to spider-pictures, but also in anticipation of cues indicating phobic content. This implies that increased vigilance in anticipation of highly relevant stimuli already modulates early sensory components. Finally, these results are in line with an observation from Fields and Kuperberg (2012), showing enhanced P1 amplitudes for words presented at the end of self-relevant sentences (i.e., directed toward the participant).

Main effects of sender were also observed in the P3 time window of Study 2, where lager amplitudes for the anticipation of feedback from both human senders compared to the computer were observed, while no differences were observed between the expert and the layperson. P3 amplitudes are found to be enlarged by top-down attention, while for the overall underlying mechanism of the P3, neuroinhibition is likewise discussed (Polich, 2007). Accordingly, we think that the P3 modulation reflects more attention directed to the adjectives acquiring feedback values from the human senders, in line with the suggestion that the P3 is enhanced by motivational relevance (Nieuwenhuis et al., 2005). For this communicative set-up, enhanced P3 amplitudes for putative human feedback are consistently observed at the feedback level (Schindler et al., 2015; Schindler and Kissler, 2016a, 2018), suggesting that the P3 is affected by the communicative context, both during anticipation and delivery of social feedback.

Overall, we believe, that along with other previous studies (Schindler et al., 2014; Rohr and Abdel Rahman, 2015), the present data nicely elucidate mechanisms of social context-dependence in emotional word processing.

### Limitations

It has to be noted that there were no direct statistical comparisons between Study 1 and Study 2, similar to other studies on socio-emotional influences on language (Rohr and Abdel Rahman, 2015). This was due to the imbalance of sender numbers between the studies, although both studies employed the same material. Since the two studies were not planned *a priori* to enable direct comparisons, and given that even within the single experiments, effects were not always very large, the results should be interpreted cautiously until replicated, ideally in one single experiment, varying the sender predictability. However, it should be noted that this might lower the credibility of the cover story, and to compensate one might need to implement confederates into the cover story. On the other hand, between-study comparisons with the same number of “senders,” facilitating direct comparisons, would not suffer from the credibility issue, but have less statistical power. Further, in line with our goal of studying the timing of emotion effects in different anticipated social contexts, we did not focus on a single ERP component, but tested sender and emotion modulations across the whole time range. This increases the number of statistical comparisons and can introduce false positives, a common problem in science (Ioannidis, 2005), specifically in small sample neuroscience studies (Button et al., 2013), leading to the call for more pre-registered research (Nosek et al., 2018). Nevertheless, this problem was targeted by running OMNIBUS ANOVA tests and by having, for this particular research area, relatively large sample sizes, highly similar experimental procedures, and identical stimuli. The results indicate a variability and plasticity in the appearance of emotion effects depending on the given context.

## Conclusion

The current two studies examined how anticipation of feedback through attributed social presence modulate ERP responses toward emotional and neutral words. Early emotional modulations (P1, N1 and EPN) were present only in Study 2 with clear sender-mapping, although late emotion effects (LPP) occurred in both studies. This indicates, that the timing of emotion effects and, possibly, the lexical access to emotional words depends on the (social) context. These findings specify the dynamics of emotional language processing, where social relevance modulates anticipatory brain responses in a specific way, leading to early attentional effects for relevant context and more differentiation of late evaluative processes within such a relevant context. In line with our expectations, a clear sender-mapping led to modulations of the P1 and P3 components, indicating increased visual attention toward relevant human senders, being initially largest for anticipating “expert”-feedback. Further, in the LPP time window, interactions of sender and emotion validate a stronger relevance of emotional adjectives when anticipating predictable human feedback. As virtual communication, including virtual feedback from physically distant and unknown senders whose identity is inferred rather than perceived, has become reality for many people worldwide, these findings are highly relevant for the fields of social neuroscience, language, and emotion processing.

## Author Contributions

JK, SS, and RV conceived the experiment and wrote the manuscript. SS and RV collected the electrophysiological data. SS analyzed the data.

## Conflict of Interest Statement

The authors declare that the research was conducted in the absence of any commercial or financial relationships that could be construed as a potential conflict of interest. The reviewer TF declared a past co-authorship with one of the authors JK to the handling Editor.

## Footnotes

1 www.neurobehavioralsystems.com

2 www.biosemi.com

3 www.besa.de

4 Please note that initially only an parieto-occipital sensor cluster was used for the P1. We included two more lateralized clusters as suggested by a reviewer to additionally capture possible early emotion effects which were visible in the topographical differences.
